# The role of tibialis posterior fatigue on foot kinematics during walking

**DOI:** 10.1186/1757-1146-3-6

**Published:** 2010-04-20

**Authors:** Michael B Pohl, Melissa Rabbito, Reed Ferber

**Affiliations:** 1 Running Injury Clinic, Faculty of Kinesiology, University of Calgary, Calgary, AB, Canada; 2 Faculty of Nursing, University of Calgary, Calgary, AB, Canada

## Abstract

**Background:**

The purpose of this study was to investigate the effect of localised tibialis posterior muscle fatigue on foot kinematics during walking. It was hypothesised that following fatigue, subjects would demonstrate greater forefoot and rearfoot motion during walking. It was also postulated that the magnitude of the change in rearfoot motion would be associated with standing anatomical rearfoot posture.

**Methods:**

Twenty-nine subjects underwent an exercise fatigue protocol aimed at reducing the force output of tibialis posterior. An eight camera motion analysis system was used to evaluate 3D foot kinematics during treadmill walking both pre- and post-fatigue. The anatomical rearfoot angle was measured during standing prior to the fatigue protocol using a goniometer.

**Results:**

Peak rearfoot eversion remained unchanged following the fatigue protocol. Although increases in rearfoot eversion excursion were observed following fatigue, these changes were of a magnitude of questionable clinical significance (<1.0°). The magnitude of the change in rearfoot eversion due to fatigue was not associated with the anatomical measurement of standing rearfoot angle. No substantial changes in forefoot kinematics were observed following the fatigue protocol.

**Conclusions:**

These data indicate that reduced force output of the tibialis posterior muscle did not alter rearfoot and forefoot motion during gait. The anatomical structure of the rearfoot was not associated with the dependence of muscular activity that an individual requires to maintain normal rearfoot kinematics during gait.

## Background

The structural integrity of the foot during gait is believed to be a combination of bony, ligamentous and muscular support [1-4]. Although cadaveric studies have demonstrated that muscles assist in maintaining both rearfoot and midfoot posture [4,5], little research has been conducted regarding the in-vivo contribution of muscular activity to controlling foot pronation during gait. The tibialis posterior is believed to play a key role as an invertor of the rearfoot [6] in addition to providing dynamic support across the midfoot [4,5]. The importance of the tibialis posterior has been highlighted by biomechanical research conducted on patients with posterior tibialis tendon dysfunction (PTTD). Studies conducted using multi-segment foot models showed that patients with PTTD demonstrated foot kinematics consistent with an excessively pronated foot during gait [7-9]. In particular, PTTD patients exhibited greater and prolonged rearfoot eversion and forefoot dorsiflexion, in addition to greater forefoot abduction, compared to controls. However, the contribution that tibialis posterior plays in controlling pronation in healthy individuals has received little attention.

One method of assessing a muscle's contribution to a specific movement pattern is via fatigue-inducing exercise of that muscle. For example, Christina et al. [10] showed that localised fatigue of the invertors resulted in a trend towards greater rearfoot eversion during running. In the afore mentioned study, fatigue of the invertors was achieved using open chain resisted supination exercises. Research has shown that selective activation of tibialis posterior was better achieved using closed chain resisted foot adduction as opposed to open chain supination [11]. Therefore, to better understand the role of tibialis posterior fatigue on foot mechanics it seems prudent to use an exercise that more selectively activates this muscle.

While rearfoot motion during gait has received much attention in the literature, its relationship with anatomical structure remains unclear. For instance, the standing rearfoot angle has been shown to be associated with rearfoot eversion during walking [12]. In contrast, Cornwall and McPoil [13] showed no relationship between static and dynamic rearfoot motion. The conflicting findings may be due to neglecting the role of muscular support when studying the relationship between the static and dynamic behaviour of the rearfoot. For example, subjects with a pronated foot posture have been shown to exhibit increased tibialis posterior activity compared to those with a normal foot structure [14]. It may be that individuals with structural deficiencies such as excessive rearfoot valgus, rely more heavily on muscular contribution to control rearfoot kinematics during gait. Thus, it might be expected that these subjects would undergo greater changes in rearfoot kinematics following fatiguing exercise of a major invertor muscle.

The purpose of this study was to examine the effect of localised tibialis posterior muscle fatigue on foot kinematics during walking. It was hypothesised that following a bout of fatigue-inducing exercise subjects would demonstrate greater and prolonged rearfoot eversion and forefoot dorsiflexion, as well as greater forefoot abduction. A secondary aim was to understand whether the magnitude of the changes in rearfoot eversion due to fatigue were associated with the anatomical measurement of standing rearfoot angle. Hence, it was hypothesised that following fatigue, subjects who underwent the greatest increases in rearfoot eversion during walking would demonstrate a greater standing rearfoot valgus posture.

## Methods

### Subjects

Based on a within group standard deviation of 5.5° (rearfoot kinematic data from Pohl et al. [15]) and an expected 15% difference between pre and post measures, α = 0.05, β = 0.80, we found that 28 subjects were needed to provide sufficient power for this study. Twenty-nine (11 males, 18 females) recreationally active subjects (mass; mean = 68.8 kg, SD = 13.5 kg) with a mean (SD) age of 27.3 years (8.1) volunteered to participate in the study. All subjects were currently free from lower extremity injury, had no prior history of surgery to the foot and lower leg, and were familiar with treadmill walking. The study was approved by the institutional ethics board, and written informed consent was obtained from all subjects.

### Data collection protocol

Prior to the gait analysis, a structural assessment of each subject's rearfoot was conducted once by a single experienced athletic therapist. With the subject lying prone, lines were drawn bisecting the lower third of the leg and the calcaneus. The relaxed standing rearfoot angle, defined as the calcaneus relative to the tibia, was then measured using a goniometer with the feet placed bi-acromial width apart [16].

Following the structural measurement, three-dimensional kinematic data were then collected for all subjects walking on a treadmill both prior to, and following, fatigue-inducing exercise of the tibialis posterior muscle of the right limb. Seventeen reflective markers (9 mm diameter) were attached to the skin of the forefoot, rearfoot and shank of the right limb [17]. An additional marker was placed on the dorsal aspect of the phalanx. Marker trajectory data were collected at 120 Hz using an eight camera motion analysis system (Vicon Motion Systems Ltd, Oxford, UK) arranged around a treadmill (StarTrac, Irvine, USA). Prior to commencement of the walking trials, a static calibration trial was recorded: subjects assumed a relaxed standing position with their feet positioned 0.30 m apart and toes pointing straight ahead. Subjects then completed treadmill walking at 1.1 ms^-1 ^while ten footfalls were collected to represent the "pre-fatigue" (PRE) condition. The marker-base attachment on the first metatarsal head was drawn around and then subsequently removed during the fatigue protocol. None of the other markers were removed. Upon completion of the fatigue exercise protocol, the first metatarsal base/marker was replaced in the same location using the outline and subjects immediately mounted (within 15 seconds) the moving treadmill to complete the "post-fatigue" (POST) walk.

### Fatigue protocol

**Figure 1:**
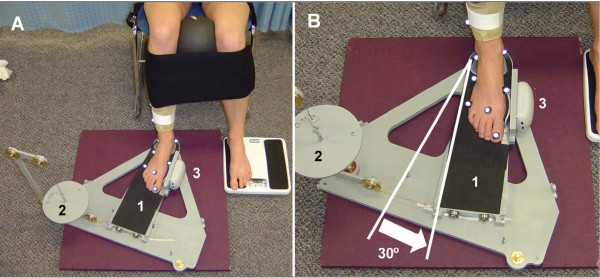
**Setup for the fatigue-inducing exercise and measurement of MVCs**. The complete setup is shown in (A) with the subject strapped into a chair with ball placement and leg straps included. The foot is positioned on a sliding foot plate (1) and foot adduction is achieved by the subject pushing their 1^st ^metatarsal head against the dynamometer (3). The MVCs were measured by locking the foot plate while the subject pushed isometrically against the dynamometer. A pulley system that allowed the placement of weights (2) provided adjustable resistance while the subject performed the fatiguing exercise through a 30° range of motion (B).

**Figure 2:**
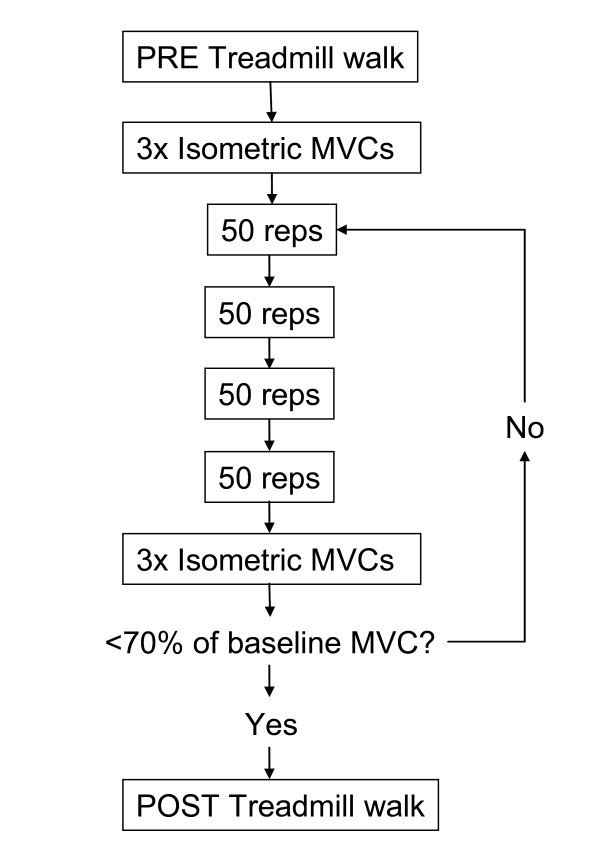
**Timeline of the experimental fatigue protocol**. Subjects who were unable to complete two consecutive sets of 50 reps had a final set of MVCs collected and proceeded directly to POST treadmill walking.

In the present study, muscle fatigue was defined as a reduction in the capacity of the muscle to perform work or generate force [10]. Tibialis posterior was fatigued using closed chain resisted foot adduction motion. This exercise was chosen since previous MRI research indicated that tibialis posterior was selectively activated during its performance compared to open chain inversion [11]. Subjects were seated in a chair while their right foot was placed in a custom built device (Figure 1a) that allowed them to perform concentric/eccentric foot adduction contractions with adjustable resistance, similar to Kulig et al. [18]. The subjects legs were stabilised by placing a ball (diameter = 16 cm) between their knees and then strapping both lower legs together and both thighs to the chair (Figure 1a). The custom built device also contained a dynamometer (Lafayette Instrument, Lafayette, USA: Model 01163)) which was fixed against the 1^st ^metatarsal head of the subject to enable the measurement of a maximal voluntary contraction (MVC) during isometric foot adduction. Prior to the commencement of the fatigue-inducing exercise, the mean of three MVC trials were taken to represent force output for the PRE conditions. Then with the ankle positioned in 20° plantarflexion, subjects performed sets of 50 concentric/eccentric contractions at 50% MVC through a 30° range of motion (Figure 1b). The subjects were allowed 10 seconds of rest between each set and after every four sets, MVCs were performed (Figure 2). The sets were continued until subjects MVCs had dropped below 70% of the PRE values or they were unable to complete two consecutive sets. A final set of MVCs were taken immediately following the post-fatigue walk (within 2 minutes) to determine whether subjects had recovered in terms of force output during the walking trial.

### Data reduction

Ten foot falls for the PRE and POST kinematic walking data were selected for analysis. Raw marker trajectory data were filtered using a fourth order low-pass Butterworth filter at 12 Hz. Visual3D software (C-motion Inc, Rockville, USA) was used to create anatomical co-ordinate systems for the shank, rearfoot and forefoot, and calculate three-dimensional segment angles [15]. All segment angles were defined as motion measured relative to the next most proximal segment [7,9]. All kinematics were analysed for the stance phase and normalised to 101 data points. The stance phase was determined using kinematic marker data. Initial contact (IC) and toe off (TO) were identified using a velocity-based algorithm [19] applied to the posterior calcaneal and dorsal phalanx markers respectively. Custom Labview (National Instruments Corp, Austin, USA) software was used to extract the following kinematic variables of interest for each subject: rearfoot peak eversion (EVE), rearfoot EVE excursion, time to peak rearfoot EVE, forefoot peak dorsiflexion (DF), forefoot DF excursion, time to peak forefoot DF, forefoot peak abduction (ABD). Peak values were defined as the peak angle that occurred during the stance phase. Excursion was defined as the angular displacement between IC and the peak value.

### Data analysis

Group descriptive statistics were calculated for each variable for both PRE and POST fatigue conditions. Paired sample t-tests were conducted for the kinematic variables of interest for between-condition statistical comparison. The relationship between the anatomical standing rearfoot angle and the changes in rearfoot kinematics following fatigue was examined using a pearson-product moment correlation. Significance for all tests was set at an alpha level of *P *< 0.05 and all analyses were undertaken using SPSS 15.0 (SPSS Inc, Chicago, USA). To examine the reliability of the kinematic walking data five subjects completed two within-day walking sessions. All markers remained on the subject with the exception of the first metatarsal head, which as described earlier, was removed following the first trial and subsequently replaced for the second trial. The between-session kinematic curve offsets for rearfoot frontal, forefoot sagittal and transverse plane motion were assessed using average root mean squared error (RMSE). The RMSE was quantified in degrees, the same unit of measurement used for the pre-post fatigue analyses.

## Results

### Force output

The mean (SD) baseline isometric force output was measured at 66.2 N (28.3). Following the fatiguing exercise protocol the mean MVC force output dropped to 44.6 N (21.8) which equated to 67% of the pre-fatigue baseline value. Eight subjects failed to drop below the predetermined force output of 70% baseline MVC. However, these subjects were still included in the analysis since they were unable to complete two consecutive sets due to fatigue. Further, all eight subjects experienced at least a 21% reduction in force output (MVC was less than 79% of the baseline value). Immediately following the post-fatigue walk, force output had increased slightly to be 80% of the baseline value.

### Kinematics

The reliability analysis performed on the subset of five subjects revealed average RMSE of 0.9°, 1.1° and 0.6° for rearfoot frontal, forefoot sagittal and forefoot transverse plane motion respectively.

**Figure 3:**
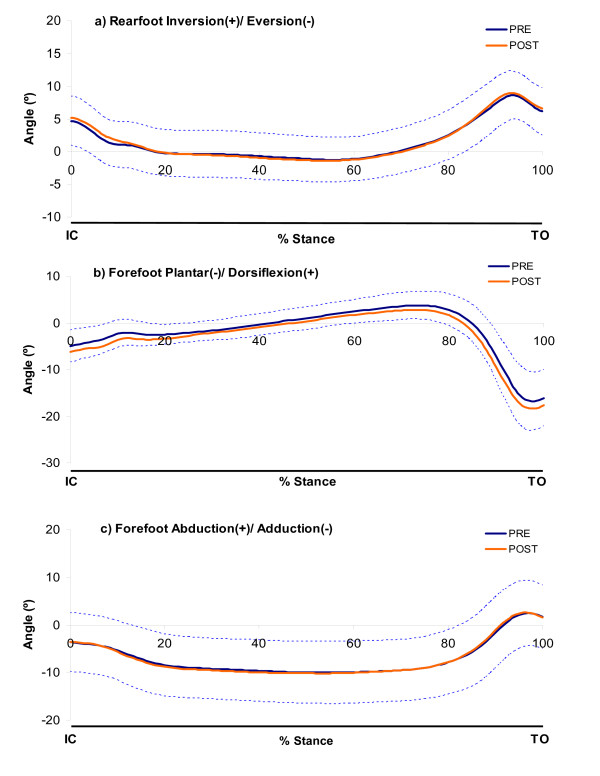
**Ensemble mean (SD) kinematic curves for both pre- and post-fatigue of rearfoot and forefoot motion**. SD is only shown for the PRE condition to improve clarity of the charts.

Mean ensemble kinematic curves of the rearfoot (relative to the shank) and the forefoot (relative to the rearfoot) are shown in Figure 3. The mean (SD) values for all kinematic variables of interest are presented in Table 1. Following fatiguing exercise of tibialis posterior, there was a significantly greater amount of rearfoot EVE excursion from the pre-fatigue condition (0.7°). There were no significant differences in rearfoot peak EVE or the time to peak EVE following the fatigue protocol.

**Table 1:** Group mean (SD) rearfoot and forefoot kinematic variables for the pre- and post-fatigue conditions.

Variable	PRE	POST	Mean Diff.	95% CI	Effect size^#^	*P*
Rearfoot peak EVE (°)	-1.8 (3.4)	-2.0 (3.4)	0.2	0.0 to 0.5	0.51	0.06
Rearfoot EVE excursion (°)	-6.5 (1.9)	-7.2 (1.8)	0.7	0.3 to 1.0	1.04	0.00*
Time to peak EVE (% stance)	44.9 (11.4)	46.7 (10.8)	-1.8	-4.7 to 1.0	0.34	0.20
Forefoot peak DF (°)	4.3 (2.8)	3.5 (2.8)	0.8	0.0 to 1.7	0.53	0.06
Forefoot DF excursion (°)	9.3 (3.0)	9.7 (3.1)	-0.5	-0.9 to 0.1	0.44	0.10
Time to peak DF (% stance)	70.4 (8.1)	69.3 (9.3)	1.2	-1.4 to 3.7	0.25	0.35
Forefoot peak ABD (°)	-10.4 (6.5)	-10.7 (6.3)	0.3	0.0 to 0.6	0.57	0.04*

*Indicates significant difference between PRE and POST (*P *< 0.05). 95% CI - 95% confidence interval of the difference. ^#^Cohen's *d *calculation.

In terms of the forefoot, the only significant change following fatigue was found for forefoot peak ABD. Although statistically significant, the increase in peak forefoot ABD was only 0.3° compared to the pre-fatigue condition. There were no significant changes in forefoot peak DF, DF excursion, or time to peak DF.

**Figure 4:**
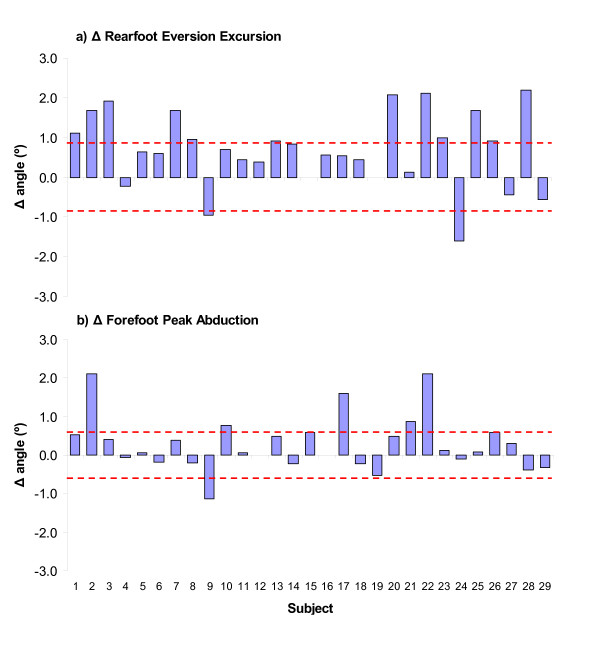
**Changes in rearfoot and forefoot kinematics of each subject following the fatigue protocol (n = 29)**. Positive bars indicate increases in the hypothesised direction (eversion and abduction) and negative bars reductions. The dashed lines indicate the precision of the measurement as determined by the within-day reliability analysis.

The individual changes in foot kinematics are presented for the variables that were significantly altered following the fatiguing exercise (Figure 4). Twenty-two out of the total 29 (76%) subjects had greater rearfoot EVE excursion following fatigue. However, only 12 of these subjects demonstrated kinematic changes that exceeded the magnitude of the within-day precision error. Of the 17 subjects (58%) that had an increase in peak forefoot ABD following the fatigue protocol, only 5 underwent increases that exceeded the magnitude of the precision error.

### Structure and kinematics

The mean (SD) anatomical standing rearfoot angle for all subjects was measured at 6.8° (2.7°) of eversion. The standing rearfoot angle was poorly correlated with the kinematic changes in both peak rearfoot eversion (r = -0.19, *P *= 0.32) and excursion (*r *= -0.28, *P *= 0.15) following fatigue.

## Discussion

The purpose of this study was to examine the changes in foot kinematics during walking following fatigue of the tibialis posterior muscle. In contrast with our original hypotheses, the results suggested that fatiguing exercise did not induce substantial changes in the magnitude or timing of rearfoot frontal plane kinematics during walking. Additonally, visual inspection of the kinematic curves (Figure 3) revealed no apparent differences throughout any part of the stance phase. There appeared to be no relationship between the static anatomical structure of the rearfoot with the magnitude of the change in rearfoot kinematics following fatigue. Contrary to our expectations, forefoot sagittal and transverse plane kinematic variables also remained unaffected following fatigue.

To investigate the relationship between tibialis posterior fatigue and foot kinematics a protocol for reducing the force output of this muscle was developed. The results indicate that fatigue protocol was successful in reducing the isometric force by over 30%, and that this force attenuation remained following the post-fatigue walking trial. Although eight subjects did not achieve the targeted 30% reduction in force production, they did all achieve at least a 21% reduction. There was no evidence that these eight subjects differed systematically from the rest of the sample in terms of kinematic changes following fatigue. The decrements in isometric force are similar to those reported by Cheung and Ng [20] for the invertors following an exhaustive run. However, the present fatigue protocol was aimed at more selectively fatiguing tibialis posterior [11] as opposed to the invertors collectively [10], in a bid to further understand the role of this specific muscle on foot kinematics.

The main finding of this investigation was the significant increase in rearfoot eversion excursion during post-fatigue walking. Although this result was statistically significant, the mean change of 0.7° was smaller than the precision error (RMSE) of a within-day gait analysis (0.9°). Peak rearfoot eversion was also unaltered (mean change of 0.2°) following fatigue suggesting that a 20-30% decrement in tibialis posterior isometric force output did not result in any measurable changes in frontal plane rearfoot kinematics. A larger mean change in peak rearfoot eversion of 1.2° has been reported by Christina et al. [10] following fatiguing exercise of the invertors musculature. However, their study used open-chain inversion exercises, making it more likely they would attenuate the force output of all the rearfoot invertors collectively, and thus induce a larger change in rearfoot mechanics than if a single invertor (tibialis posterior) was fatigued selectively. Therefore, in the present study it is possible that other muscles such as tibialis anterior, may have compensated for the lack of tibialis posterior force production. In addition, Christina and colleagues [10] measured the changes in rearfoot kinematics during running, where ground reaction forces and joint excursions are double in magnitude compared with walking. It could be speculated that greater muscle activity is required during running and thus, greater increases in rearfoot eversion would be expected following fatigue.

The fatigue protocol did not appear to induce substantial changes in forefoot kinematics during walking. Although statistically significant, the 0.3° increase in forefoot abduction following fatigue could not be feasibly detected using motion analysis. These results are in contrast to the PTTD literature, which suggests that a deficient tibialis posterior is associated with greater forefoot abduction and dorsiflexion [7,9]. However, the PTTD patients from whom these findings were derived had advanced stage II symptoms including rearfoot valgus and forefoot abduction deformities. Although PTTD patients attempt to compensate using other muscles [21], it is possible that their structural deformities are too severe to achieve normal foot kinematics. Moreover, since the subjects used in the present investigation had relatively normal foot structure, they may have been able to successfully compensate for the tibialis posterior via the activation of other muscles. Indeed, extrinsic muscles such as tibialis anterior, flexor digitorum longus, flexor hallucis longus, peroneus longus [4] along with intrinsic foot muscles [22,23] have been shown to play a role in maintaining the structural integrity across the midfoot. Alternatively, given that the subjects in the present study did not have notable foot structural deformities, tissues such as the plantar fascia [24] and spring ligament complex [3] may have prevented alterations in forefoot kinematics occurring following the fatigue protocol.

The mean standing rearfoot angle measured in the present study is in agreement with values reported in the literature for larger samples [13,16]. Interestingly, there was a poor relationship between the standing rearfoot angle with the changes in rearfoot walking kinematics that were observed following fatigue. Therefore, subjects who had greater standing rearfoot valgus angles did not rely more on tibialis posterior to control rearfoot motion during walking. These preliminary results suggest that the anatomical structure of the foot is not associated with the dependence of muscular activity that an individual requires to maintain normal foot kinematics during gait. However, it has been discussed earlier that reduced force output of tibialis posterior may have been compensated for by other muscles. Therefore, it is possible that compensation strategies may have masked the true relationship between anatomical structure and tibialis posterior contribution. The present investigation was also limited by the fact that the standing rearfoot angle was the only structural measurement of the foot. For instance, the range of motion (ROM) of the rearfoot might influence the degree to which an individual's rearfoot eversion can change following fatigue. If the peak rearfoot eversion angle was close to their end ROM during the pre-fatigue gait, then structural restraints would prevent any further increases in eversion following fatigue. In addition, structural measures of the arch and forefoot have not been reported here. It is possible that a rigid cavus foot would rely less on muscular contribution to maintain the integrity of the arch and forefoot during walking. Indeed a recent study demonstrated that during gait, flat-arched individuals exhibit greater activity of tibialis posterior compared to those with normal arches [14]. Future studies with more comprehensive foot structure evaluations are required to understand the contributions of bony anatomy and muscular activation to foot biomechanics.

While foot adduction has been shown to be the best exercise at selectively activating tibialis posterior [11], other muscles also play a role in this movement. There is also lack of literature to indicate whether this movement can reliably activate tibilias posterior. Therefore, this study was limited in its ability to directly observe or quantify the degree of fatigue that was achieved in the tibialis posterior muscle. Future research is needed to determine how valid and realiable the fatigue protocol was in selectively fatiguing tibialis posterior. Another approach would be to utilise electromyography to quantify changes in muscle activity and fatigue [23]. Future studies using electromyography would also enable greater understanding of the compensation strategies employed by other muscles.

## Conclusions

No substantial changes in rearfoot eversion were found during walking following an exercise protocol aimed at reducing the force output of the tibialis posterior muscle. In addition, the anatomical structure of the rearfoot was not associated with the magnitude of the change in rearfoot kinematics following fatigue. Changes in forefoot kinematics were also not observed following the fatigue protocol.

## Competing interests

The authors declare that they have no competing interests.

## Authors' contributions

MBP and RF developed the rationale for the study. MBP and MR designed the study protocol, conducted the data collections and processed the data. MBP, MR and RF drafted the manuscript. All authors have read and approved the final manuscript.
